# Self-Monitoring in Speech Production: Comprehending the Conflict Between Conflict- and Comprehension-Based Accounts

**DOI:** 10.5334/joc.118

**Published:** 2020-09-03

**Authors:** Andreas Lind, Robert J. Hartsuiker

**Affiliations:** 1 Lund University Cognitive Science, Lund University, SE; 2 Department of Experimental Psychology, Ghent University, BE

Verbal self-monitoring is the set of processes speakers use to inspect their own speech and to intervene when trouble arises. Theories differ in the modality and manner in which they propose that monitoring is achieved. There are a number of external feedback channels that can potentially be used for self-monitoring: auditory and bone conducted feedback reach the cochlea and allow us to hear our own voice,^1^ proprioceptive feedback gives us information about the position of the articulators, and tactile feedback gives us information about oral surfaces hitting each other. The social effects of our words on our interlocutors, cognized via sound and vision, could also be considered a feedback channel (see e.g. Levelt, 1989; Postma, 2000).

In addition to these external monitoring channels, several other types of channel have been proposed. Most prominently, an internal form of monitoring has been suggested to account for error-monitoring data that cannot be easily explained by appealing only to external channels (see Gauvin & Hartsuiker, 2020, pp. 2 and Roelofs, 2020a, pp. 2, for a summary of this data). While apparently distinct from inner speech, the type of language-based reflection that we can carry out silently “inside our heads” (see e.g., Alderson-Day & Fernyhough, 2015), the nature of this internal monitoring channel is up for much debate. Roelofs’s comprehension-based model WEAVER++ assumes that internal monitoring is achieved by the regular speech comprehension system forwarding the output of the word-form encoding process, the phonological word, to the monitor, which compares it with the selected production representations (e.g., Roelofs, 2004; 2005; 2020a). The conflict monitoring account championed by Nozari (2020) and Gauvin and Hartsuiker (2020), on the other hand, postulates that internal monitoring is carried out by monitoring the amount of conflict in either lemma or phoneme selection. An important set of questions for self-monitoring thus concern the internal and external channels that the speaker routinely employs for the purpose of monitoring. Do speakers primarily focus on only one or a few channels or do they rely on an entire repertoire of such channels? Given that speakers can monitor a multitude of linguistic and social-communicative levels, does the answer to the previous question depend on the level that is monitored? And how does each monitoring level work precisely?

This special issue is based on the symposium “Self-monitoring in Speech Production” that we organized at the 2017 conference of the European Society for Cognitive Psychology (ESCoP), held in Potsdam, Germany. The symposium featured contributions from Hanna Gauvin, Nazbanou Nozari, Gregory Hickok, and Martin Pickering. The former two researchers, along with Ardi Roelofs, have provided the contributions to this special issue. The symposium speakers represented and debated a range of theoretical positions and foci and were explicitly asked to think about whether and how such different positions may converge (or not) and which open questions are most salient. The collection of papers in this special issue continues this discussion.

Specifically, in the first half of their paper in this issue, Gauvin and Hartsuiker (2020) present an overview of the most prominent current models of speech monitoring. These models differ in the importance and roles they assign to all the different potential channels, and in how the channels are thought to interact with each other in verbal monitoring. For example, the conflict monitoring model (Nozari et al., 2011) proposes that monitoring is primarily performed without any external feedback, while the other models all maintain that auditory feedback is crucial. Gauvin and Hartsuiker point out several lacunae of current proposals. For one, no model has an adequate account of error detection in *other-perception*. Additionally, most accounts stop at the moment of error detection; however, what are the mechanisms by which detection of an error or other problems leads to an adjustment of one’s behaviour, such as a correction? Gauvin and Hartsuiker explore one avenue, according to which conflict detection indirectly boosts the strength of feedforward encoding processes. They have implemented this mechanism “on top” of Nozari et al.’s conflict monitoring model and show by simulation how an error-related boost can suppress semantic errors.

The remaining three papers are an exchange between Roelofs and Nozari where they cut to the core of a number of points of disagreement between their respective models. In the target article of Roelofs (2020a), the author makes a case for a specific type of comprehension-based monitoring model which is based on the WEAVER++ model of speech production. He argues that the theoretical and empirical arguments that have been raised against a comprehension-based account over the years, many of which have come from, or have been reiterated by, Nozari and colleagues (Nozari et al., 2011; Nozari & Novick, 2017; see also e.g., Gauvin et al., 2016; Huettig & Hartsuiker, 2010; Vigliocco & Hartsuiker, 2002), do not, in fact, undermine such a model. One of these arguments is for instance that there should be interference between representations of speech processed through an internal and an external channel with only a short temporal lag. Vigliocco and Hartsuiker (2002) argued that such near-synchronous representations in the speech comprehension system should lead to cross-talk, or even the subjective experience of “echoes”. Roelofs, however, argues that this cross-talk problem can be solved by a mechanism in the WEAVER++ model that produces separate processing threads for internally and externally perceived speech. Similarly, Roelofs argues that double dissociations between comprehension and monitoring in aphasic patients are not really a problem for WEAVER++ as monitoring is not based solely on comprehension but also on a comparison process, allowing each of these processes to be selectively impaired, and that current neuroimaging data does not provide consistent evidence for a production-based and against a comprehension-based account.

In her reply to Roelofs, Nozari (2020) provides a further review of monitoring accounts and then re-examines Roelofs’s arguments in defense of a comprehension-based account. In particular, she first sketches an important property of comprehension-based monitors such as Levelt’s (1989) perceptual-loop theory: that comprehension monitoring is an attentional function, and hence deliberate, flexible, and conscious. According to Nozari, the model in Roelofs (2020a) however abandons this basic property of comprehension monitoring and can therefore be likened more to what Nozari refers to as a production-perception (or forward modeling) account. However, in Nozari’s view, Roelofs’s model lacks a number of relevant properties of production-perception accounts: in particular, such an account requires separate representations in production and perception (as is arguably the case for low level sound and syllable representations) and a process of “coordinate transform” between such representations (Guenther, 1994; Hickok, 2012). Nozari also takes issue with the notion of condition-action rules in Roelofs’s model and their motivation from the notion that monitoring is a goal of speaking. She then re-examines the evidence against comprehension monitoring that Roelofs discussed and argues that Roelofs’s dismissal of these arguments may be premature: for instance, dismissal of the “cross-talk” example seem to require that condition-action rules are invoked.

In the final contribution, Roelofs (2020b) responds to Nozari’s criticism. He argues that conscious and deliberate processing is a property of some comprehension-based models but is not a necessary feature. He also clarifies that the overall goal of speaking is of course communication, but that in his view condition-action rules are a mechanism by which the speaker can ensure that communication stays on track. He cites evidence from cognitive science and neuroscience to argue further for condition-action rules. He then turns to the arguments against comprehension monitoring again and argues that his dismissal of these arguments still holds. He concludes that comprehension-based monitoring remains a viable account of self-monitoring in speaking.

It is clear from this exchange of ideas that there is little consensus in the field yet about the most fruitful road towards a detailed understanding of verbal self-monitoring. Some of these differences seem to go back to different views of how to best model the language production system (e.g., is it a good idea to build condition-action rules into the system?) Perhaps an even more fundamental issue concerns the requirements one would have of a theory. Roelofs (2020a; b) appears to champion a single-mechanism account that relies on comprehension only, for both internal and external monitoring. Nozari (2020) rather appears to promote a multi-mechanism account, according to which the mechanism that detects errors depends on the level of speech that is being monitored. Detecting a deviation in pitch might best be explained with a forward model account, while a more viable explanation for lexical error detection could be conflict monitoring. One way of putting this is that the authors are taking different positions in the trade-off between parsimony and scope. Relatedly, regardless of whether conscious or deliberate processes are (Nozari) or are not (Roelofs) necessarily part of comprehension-based monitoring, the question remains to what extent such processes play a role in monitoring (also see Postma, 2000).

The papers presented here raise many issues for future research on monitoring, including the need to pay more attention to closely related processes, such as error-monitoring in perception (i.e., detecting that *another person* is making a speech error) and the aftermath of error detection, such as interruption, repair, or even prevention of the error by increasing guidance of the production system (Gauvin & Hartsuiker, 2020). We would like to end with raising awareness to two issues that none of the contributors explicitly address. First, while all models appear to presuppose that the starting point for speech is a pre-linguistic message, the meaning and basic structure of which seems to be taken as already fully formed as it sets the production machinery in motion (see Lind et al., 2014a; 2014b for a critique of this), monitoring accounts differ in the standard against which an utterance is judged to be correct or erroneous. Such a standard could be a forward model of a syllable or speech segment (in forward model accounts), a lemma representation (in WEAVER++), or even the prelinguistic message itself (in the original Perceptual Loop Theory, e.g., Levelt, 1989). Crucially, Nozari et al.’s (2011) conflict monitoring theory does away with a comparison with a standard, as error signals are here based on a measure of processing dynamics within the production system. Thus, the monitoring mechanism has only statistical information about the occurrence of an error to go on (“given the current amount of conflict, an error is likely to have happened”). This, however, would seem to imply that the repair mechanism is in fact blind to whether or not an error has occurred, what was wrong with the utterance, and perhaps even which word was an error.

Second, as we have seen, there are many external monitoring channels (auditory, bone conduction, proprioceptive, tactile, visual, and social), several of which have been shown to play a role in different types of monitoring situation. But no model seems to find a use for all of these (as we have seen, Nozari speculates that multiple mechanisms are likely used to achieve monitoring, but she does not specify which channels are involved in which way). One might wonder whether monitoring for speech errors has analogies with processes like depth perception, where perceivers use both binocular and a range of monocular cues like shading, occlusion, spatial frequencies, and so on. Additionally, as in depth perception one can then ask whether information from each cue has a similar or different weight, and whether some cues only come into play, or are given greater weight, when other cues are absent or noisy (as was suggested already by Ladefoged, 1967; note also that Hartsuiker & Kolk, 2001, proposed a solution for the case of a dual-loop comprehension-based model). In our view, addressing how the multitude of potential cues play out during verbal self-monitoring, and what standards they are, or are not, measured against, remain important questions both for the accounts discussed in this special issue, and also more generally for the future of research into speech and monitoring.

## Footnotes

The issue of how bone conducted feedback contributes to self-monitoring and self-recognition of one’s own voice has, to our knowledge, not been explored but is an interesting issue, not least because bone conduction enhances lower frequencies of the voice during self-perception, see e.g., Reinfeldt et al. (2010).

## References

[1] Alderson-Day, B., & Fernyhough, C. (2015). Inner speech: Development, cognitive functions, phenomenology, and neurobiology. Psychological Bulletin, 141, 931–965. DOI: 10.1037/bul0000021 26011789 PMC4538954

[2] Gauvin, H. S., De Baene, W., Brass, M., & Hartsuiker, R. J. (2016). Conflict monitoring in speech processing: An fMRI study of error detection in speech production and perception. Neuroimage, 126, 96–105. DOI: 10.1016/j.neuroimage.2015.11.037 26608243

[3] Gauvin, H. S., & Hartsuiker, R. J. (2020). Towards a new model of verbal monitoring. Journal of Cognition. 3(1): 17, pp. 1–37. DOI: 10.5334/joc.81 PMC7473241 32944680

[4] Guenther, F. H. (1994). A neural network model of speech acquisition and motor equivalent speech production. Biological Cybernetics, 72, 43–53. DOI: 10.1007/BF00206237 7880914

[5] Hartsuiker, R. J., & Kolk, H. H. J. (2001). Error monitoring in speech production: A computational test of the perceptual loop theory. Cognitive Psychology, 42, 113–157. DOI: 10.1006/cogp.2000.0744 11259106

[6] Hickok, G. (2012). Computational neuroanatomy of speech production. Nature Reviews Neuroscience, 13, 135–145. DOI: 10.1038/nrn3158 22218206 PMC5367153

[7] Huettig, F., & Hartsuiker, R. J. (2010). Listening to yourself is like listening to others: External, but not internal, verbal self-monitoring is based on speech perception. Language and Cognitive Processes, 25, 347–374. DOI: 10.1080/01690960903046926

[8] Ladefoged, P. (1967). Three Areas of Experimental Phonetics. London: Oxford University Press.

[9] Levelt, W. J. M. (1989). Speaking. Cambridge, MA: MIT Press.

[10] Lind, A., Hall, L., Breidegard, B., Balkenius, C., & Johansson, P. (2014a). Speakers’ acceptance of real-time speech exchanges indicates that we use auditory feedback to specify the meaning of what we say. Psychological Science, 25, 1198–1205. DOI: 10.1177/0956797614529797 24777489

[11] Lind, A., Hall, L., Breidegard, B., Balkenius, C., & Johansson, P. (2014b). Auditory feedback of one’s own voice is used for high-level semantic monitoring: The “self-comprehension” hypothesis. Frontiers in Human Neuroscience, 8, Article 166 DOI: 10.3389/fnhum.2014.00166 PMC3975125 24734014

[12] Nozari, N. (2020). A comprehension- or a production-based monitor? Response to Roelofs (2020). Journal of Cognition. 3(1): 19, pp. 1–21. DOI: 10.5334/joc.102 PMC7473204 32944682

[13] Nozari, N., Dell, G. S., & Schwartz, M. F. (2011). Is comprehension necessary for error detection? A conflict-based account of monitoring in speech production. Cognitive Psychology, 63, 1–33. DOI: 10.1016/j.cogpsych.2011.05.001 21652015 PMC3135428

[14] Nozari, N., & Novick, J. (2017). Monitoring and control in language production. Current Directions in Psychological Science, 26, 403–410. DOI: 10.1177/0963721417702419

[15] Postma, A. (2000). Detection of errors during speech production: a review of speech monitoring models. Cognition, 77, 97–131. DOI: 10.1016/S0010-0277(00)00090-1 10986364

[16] Reinfeldt, S., Östli, P., Håkansson, B., & Stenfelt, S. (2010). Hearing one’s own voice during phoneme vocalization – Transmission by air and bone conduction. Journal of the Acoustical Society of America, 128, 751–762. DOI: 10.1121/1.3458855 20707445

[17] Roelofs, A. (2004). Error biases in spoken word planning and monitoring by aphasic and nonaphasic speakers: Comment on Rapp and Goldrick (2000). Psychological Review, 111, 561–572. DOI: 10.1037/0033-295X.111.2.561 15065924

[18] Roelofs, A. (2005). Spoken word planning, comprehending, and self-monitoring: Evaluation of WEAVER++ In R. J. Hartsuiker, R. Bastiaanse, A. Postma & F. Wijnen (Eds.), Phonological encoding and monitoring in normal and pathological speech (pp. 42–63). Hove, UK: Psychology Press.

[19] Roelofs, A. (2020a). Self-monitoring in speaking: In defense of a comprehension-based account. Journal of Cognition. 3(1): 18, pp. 1–13. DOI: 10.5334/joc.61 PMC7473215 32944681

[20] Roelofs, A. (2020b). On (correctly representing) comprehension-based monitoring in speaking: Rejoinder to Nozari (2020). Journal of Cognition. 3(1): 20, pp. 1–7. DOI: 10.5334/joc.112 PMC7473236 32944683

[21] Vigliocco, G., & Hartsuiker, R. J. (2002). The interplay of meaning, sound and syntax in sentence production. Psychological Bulletin, 128, 442–472. DOI: 10.1037/0033-2909.128.3.442 12002697

